# Leveraging Large Language Models for Automating Outpatients’ Message Classifications of Electronic Medical Records

**DOI:** 10.3390/healthcare13233052

**Published:** 2025-11-25

**Authors:** Amima Shifa, G. G. Md. Nawaz Ali, Roopa Foulger

**Affiliations:** 1 Department of Computer Science and Information Systems, Bradley University, Peoria, IL 61625, USA; amimashifa@mail.bradley.edu; 2 Digital Innovation Development, OSF Innovation, Peoria, IL 61603, USA; roopa.foulger@osfhealthcare.org

**Keywords:** large language models, message classification, hospital data, natural language processing, healthcare

## Abstract

**Background:** The widespread adoption of digital systems in healthcare has produced large volumes of unstructured text data, including outpatient messages sent through electronic medical record (EMR) portals. Efficient classification of these messages is essential for improving workflow automation and enabling timely clinical responses. **Methods:** This study investigates the use of large language models (LLMs) for classifying real-world outpatient messages collected from a healthcare system in central Illinois. We compare general-purpose (GPT-4o) and domain-specific (BioBERT and ClinicalBERT) models, evaluating both fine-tuned and few-shot configurations against a TF-IDF + Logistic Regression baseline. Experiments were performed under a HIPAA-compliant environment using de-identified and physician-labeled data. **Results and Conclusions:** Fine-tuned GPT-4o achieved 97.5% accuracy in urgency detection and 97.8% in full message classification, outperforming BioBERT and ClinicalBERT. These results demonstrate the feasibility and validity of applying modern LLMs to outpatient communication triage while ensuring both interpretability and privacy compliance.

## 1. Introduction

The modern healthcare system produces a massive volume of data every day, with hospitals estimated to generate over 50 petabytes of information annually [1]. Up to 80% of this data is unstructured, comprising clinical notes, patient–provider communications, administrative records, and other narrative content [2]. This unstructured data poses substantial challenges for traditional data processing methods due to its irregular format, medical jargon, and contextual ambiguity. Efficient management and classification of healthcare data can play a crucial role in improving operational workflows, enabling more responsive patient care, and reducing administrative burden. The integration of artificial intelligence (AI) into healthcare, particularly through machine learning and LLMs, has shown significant potential in automating various tasks and enhancing communication pathways within clinical settings [3]. For instance, identifying urgent messages from patients or distinguishing between appointment requests and medical questions promptly can directly impact healthcare outcomes and staff workload [4]. Studies have indicated that implementing machine learning approaches can optimize workflows and provide substantial benefits regarding patient care management [5,6,7]. Our work focuses on this important direction as we seek to develop strategies that enhance the efficiency and responsiveness of healthcare services.

For text classification in healthcare applications, traditional approaches such as rule-based systems and classical machine learning methods have been used widely but often lack scalability and adaptability. Prior studies have explored the use of support vector machines (SVMs), decision trees, and ensemble learning techniques for clinical text classification [8,9]. However, these approaches require extensive feature engineering and struggle with contextual variations.

Deep learning-based methods, particularly those leveraging recurrent neural networks (RNNs) and convolutional neural networks (CNNs), have demonstrated improved performance in classifying electronic health records (EHRs) [10]. More recently, transformer-based models, such as BERT and its domain-specific adaptations like BioBERT and ClinicalBERT, have achieved state-of-the-art results in medical NLP tasks [11,12]. These models leverage contextual embeddings, significantly improving accuracy over static word embeddings.

The past two years have also seen the emergence of specialised clinical-triage LLMs, such as MedAlpaca [13] and GatorTron [14], which extend transformer architectures to large-scale biomedical corpora. MedAlpaca focuses on instruction tuning for medical reasoning and patient interaction tasks, while GatorTron, trained in more than 90 billion words from clinical narratives, has demonstrated strong performance on tasks ranging from concept extraction to clinical question answering. These advances highlight the growing maturity of domain-specific LLMs for triage-related applications, providing an important benchmark for evaluating new models.

Despite the effectiveness of domain-specific Large Language Models (LLMs), recent advances in general-purpose models like GPT-4 have shown remarkable adaptability across various NLP tasks, including medical text classification [15]. Studies comparing specialized and general LLMs indicate that fine-tuning large generalist models on domain-specific data can outperform specialized models in classification tasks [16]. Our study builds upon this research by comparing several LLMs including BioBERT [11], ClinicalBERT [12], and GPT-based [17] models for hospital message classification.

Recent developments in NLP, particularly through the rise of LLMs, have enabled more accurate and scalable solutions for handling such complex, text-heavy tasks. These models have proven effective across domains, including answering clinical examination questions [18], generating radiology and discharge summaries [19], and supporting administrative workflows [20].

However, healthcare applications of LLMs must contend with unique challenges. These include domain-specific vocabulary, strict privacy constraints, variations in communication styles between patients and clinicians, and the high cost of misclassifications in sensitive contexts. Models must not only understand medical concepts but also generalize across diverse patient populations and communication settings.

This paper focuses on the real-world implementation of LLMs for classifying outpatient messages exchanged through the EMR platform. To ensure terminological consistency, the term *outpatient messages* is used throughout this study to describe patient-initiated communications sent via the EMR portal. These messages typically include appointment requests, medication questions, and clinical updates requiring provider review.

Our limited dataset was collected from a hospital system based in central Illinois and was carefully de-identified to ensure compliance with HIPAA regulations [21]. We evaluated the performance of domain-specific models, BioBERT and ClinicalBERT and compared them to GPT-4o, a powerful general-purpose model from OpenAI. We explore both few-shot prompting and fine-tuning strategies to determine which approach best suits the complexity and variability of clinical message classification. From a theoretical perspective, our work aligns with current frameworks in clinical informatics and human–AI collaboration, where message triage is modeled as a multi-stage decision process balancing linguistic understanding, contextual reasoning, and operational prioritization.

Our major contributions are summarized below.

- We introduce a practical framework for classifying outpatient messages using both domain-specific and general-purpose LLMs.
- We conduct a comparative evaluation of BioBERT, ClinicalBERT, and GPT-4o across urgency detection and multi-label categorization tasks against a baseline Logistic Regression model.
- We present insights into the fine-tuning process of BioBERT, ClinicalBERT, and GPT-4o within a secure hospital cloud environment, highlighting accuracy gains, ethical safeguards, and deployment considerations.

The rest of the paper is organized as follows. Section 2 lays the foundation of this work. The description of used LLM models and data set are stated in Section 3. Comprehensive results and analysis are stated in Section 4, followed by a discussion summary in Section 5. We finally conclude this paper in Section 6.

## 2. Motivation

In today’s fast-paced healthcare environment, timely communication between patients and providers is crucial. Every day, healthcare systems receive a high volume of patient messages ranging from appointment requests to urgent medical concerns. However, managing this influx of messages efficiently poses a significant challenge. The current system relies on significant human involvement for message management. Although this ensures careful attention, it also makes it difficult to prioritize messages consistently, placing additional strain on providers.

To address this challenge, we propose an innovative, AI-driven solution that leverages a customized OpenAI model (fine-tuned ChatGPT-4o) to automate message classification and response generation. Our goal is to develop an intelligent system that not only categorizes messages based on urgency and content but also suggests appropriate responses or routes them to the right healthcare professionals. By integrating this system with the Hospital Community Connect (HCC) platform, we aim to create a seamless and efficient communication workflow that enhances patient engagement and provider efficiency.

The motivation behind this project is twofold. First, it seeks to alleviate the burden on healthcare workers, allowing them to focus on direct patient care rather than administrative tasks. Second, it aims to ensure that urgent patient concerns are addressed promptly, improving health outcomes and patient satisfaction. Given the limitations of off-the-shelf AI models in handling nuanced, multi-class classification tasks in healthcare, we plan to build a domain-specific, fine-tuned model trained on real-world patient messages. This approach will enable our system to achieve higher accuracy and reliability in classifying and responding to messages. Our motivation is both practical and visionary:

- **Operational Impact:** By automating classification, we can reduce administrative workload and improve response times.
- **Clinical Relevance:** Accurate triage ensures that critical health issues are identified and escalated without delay.
- **Technological Advancement:** We test the hypothesis that a fine-tuned general-purpose LLM can outperform existing domain-specific models in nuanced, multi-label classification tasks.

By leveraging cutting-edge natural language processing techniques and AI-driven automation, this project has the potential to revolutionize patient–provider communication. It represents a step towards a more responsive, efficient, and patient-centered healthcare system where technology empowers providers and patients to engage more effectively. We believe our work will advance the application of AI in healthcare and set a precedent for future AI-driven innovations in clinical communication and digital health solutions.

**Research Hypotheses:**

**Hypothesis 1.**  *A fine-tuned general-purpose LLM (GPT-4o) can outperform domain-specific models (BioBERT, ClinicalBERT) in nuanced, multi-label outpatient message classification.*

**Hypothesis 2.**  *Fine-tuning improves classification accuracy compared with few-shot prompting for clinical text.*

## 3. Methodology

### 3.1. Overview

Several large language models were evaluated, including BioBERT, ClinicalBERT, and GPT-4o. Each model was tested with and without fine-tuning to determine which achieved the best performance in classifying healthcare messages across three tasks: urgency detection, category identification, and multi-label classification. To establish a benchmark, we compared the LLMs against a baseline Logistic Regression model. This enabled a fair assessment of whether the added complexity of LLMs was justified over simpler, lower-cost methods. The rationale for employing multiple message-classification subtasks stems from the nature of real clinical communication. Patient messages often contain multiple intents and vary in urgency. An effective triage system must identify both the immediacy of response and the specific type of inquiry- administrative, clinical, or logistical.

This three-step classification structure comprising urgency detection, single versus multiple intent identification, and message category labeling was developed in consultation with hospital physicians and nursing staff. The framework directly reflects how clinicians and triage nurses prioritize and route messages in everyday hospital operations. In practice, staff first determine whether a message is urgent, then assess whether it contains one or multiple distinct issues, and finally assign it to the relevant clinical or administrative category. Aligning our classification process with this existing workflow ensures that model predictions are interpretable and clinically meaningful, maintaining consistency with established hospital communication procedures.

Urgency classification is vital for ensuring timely responses to critical issues that could impact patient outcomes, while category identification enables accurate routing of messages to the proper department or clinician. Table 1 illustrates de-identified examples of patient messages, showing urgency level, message type, and categories.

**Figure 1:**
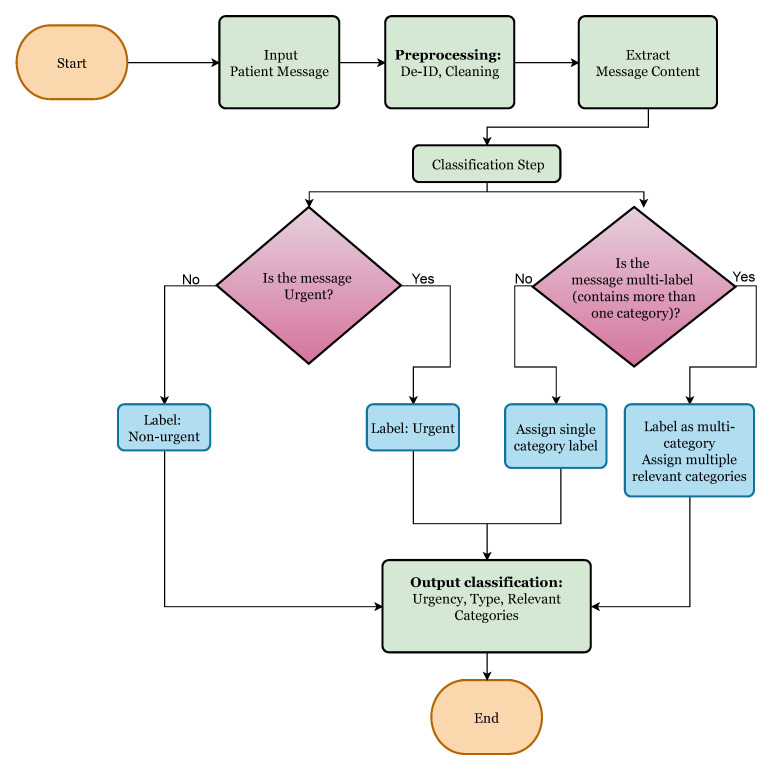
Flowchart for message-classification decision-making.

To operationalize this classification pipeline, we designed a decision-based flowchart, Figure 1 to guide how a message is processed. The process begins with evaluating whether the message is urgent or not urgent after categorizing the urgency; the next step assesses whether the message involves a single issue or multiple distinct concerns. Finally, based on content, the model assigns one or more labels from the pre-defined category set. All data were obtained from a single healthcare system in central Illinois. Consequently, model generalizability to other institutions or regional communication styles remains unverified; this limitation is discussed further in Section 5.

### 3.2. Dataset Description

**Figure 2:**
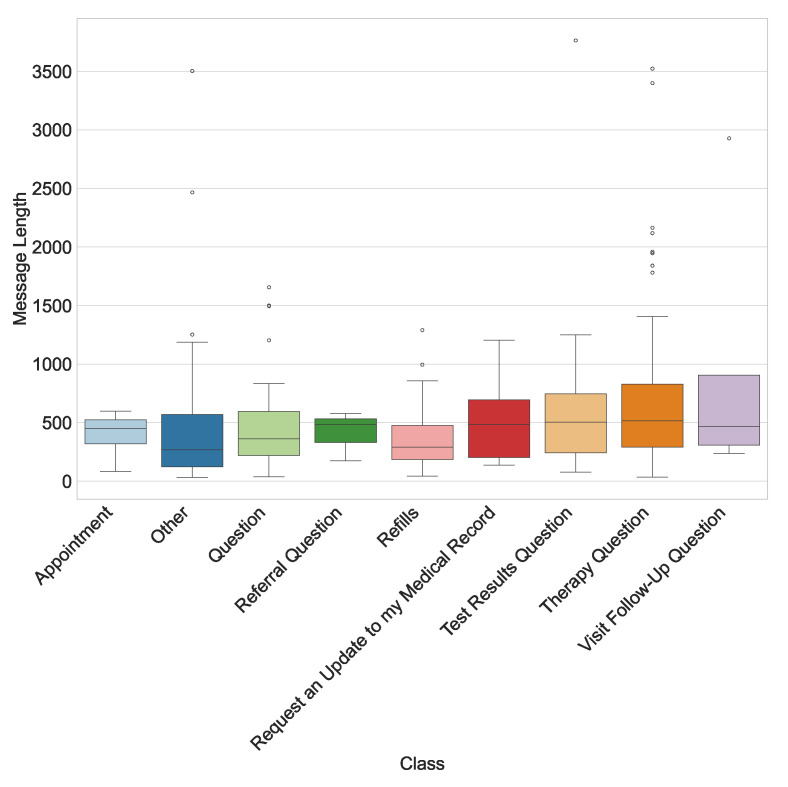
Distribution of annotated message categories in the dataset. The category “Question” refers to general inquiries without a specific intent, whereas “Other” includes non-clinical or misrouted messages.

**Figure 3:**
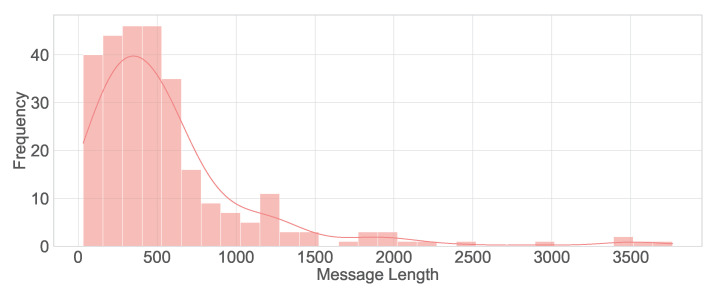
Histogram of message lengths.

**Figure 4:**
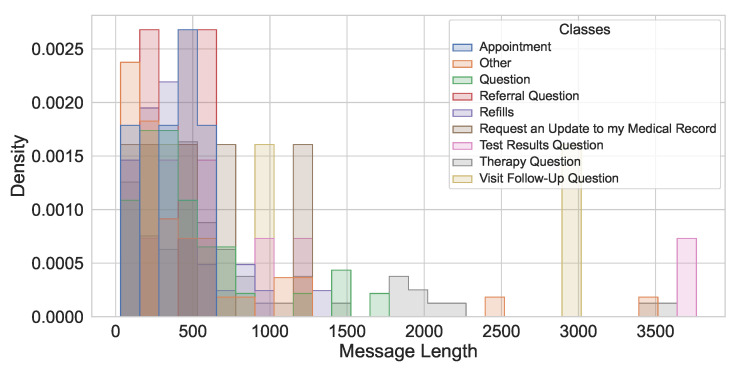
Multi-label class distribution.

**Figure 5:**
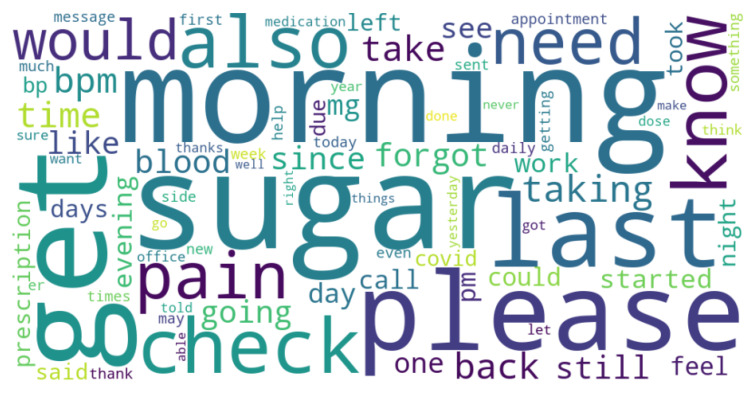
Word cloud of common terms in messages.

This study utilizes a real-world hospital dataset containing patient messages collected from an internal hospital communication system. The dataset includes textual messages related to administrative inquiries, appointment scheduling, medication refills, and clinical updates. Data preprocessing involved de-identification, so all personally identifiable information (PII) was removed to comply with HIPAA regulations. A total of 280 messages were manually labeled with the assistance of hospital physicians experienced with the outpatient communication system. This labeled data set has been used for training and testing the LLMs studied. The distribution of messages by their true classes is shown in Figure 2. The message length’s histogram shows the majority of messages are under 600 characters (Figure 3), and the multi-label message classes against their true labels are shown in Figure 4 reveals that some of the messages fall into more than one category. The word cloud depicts the most frequent words in these messages as shown in Figure 5.

To clarify the label schema, the category *Question* was used by hospital physicians for general medical inquiries that did not specify a clear medical or administrative intent, such as short open-ended queries (e.g., “Can I schedule soon?” or “Is this refill available?”). In contrast, the *Other* label represented messages that were non-clinical, irrelevant, or administrative anomalies (e.g., duplicate submissions or misrouted messages). This distinction was maintained to preserve annotation consistency and to reflect real-world message ambiguity observed in EMR communication.

During text preprocessing, messages were lowercased, punctuation was normalized, and common English stopwords were removed using the NLTK library. However, politeness markers such as “please,” “thank,” and “thanks” were intentionally retained after consultation with hospital staff. These tokens often serve as pragmatic cues of message intent and tone; for example, urgent requests tend to use more direct phrasing, whereas polite forms are common in non-urgent administrative queries. Retaining these expressions therefore preserves clinically meaningful linguistic variation.

**Dataset Statistics:**

- Total messages: **280**.
- Average message length: **100.43** words.
- Category Distribution: **9** categories were distributed unevenly throughout the dataset.

### 3.3. Modeling Approaches and Prompt Design

In this study, we adopted three complementary strategies for classifying patient portal messages: (i) a simple baseline using TF-IDF with Logistic Regression, (ii) non-fine-tuned prompting of large language models (LLMs), and (iii) fine-tuned LLMs trained on domain-specific annotated data. These approaches were selected to balance interpretability, performance, and practical deployment considerations.

**Baseline: TF-IDF + Logistic Regression**

As a lightweight benchmark, we implemented a TF-IDF vectorizer with a logistic regression classifier for two core subtasks: (i) urgency detection (binary) and (ii) single vs. multiple category classification (binary). This baseline provides a transparent, low-cost reference point against which the gains of LLM-based methods can be evaluated.

**Non-Fine-Tuned Prompting**

We constructed task-specific prompts for GPT-4o and domain-specific LLMs (BioBERT, ClinicalBERT) without fine-tuning. Each patient message was wrapped in a structured chat format with explicit instructions, ensuring that model outputs matched the exact label schema. Below we list the representative prompt templates. The prompt used for Task A (Urgency Classification) is shown in Listing 1.

**Listing 1.** Prompt used for Task A: Urgency Classification.
              {"messages": [                              {"role":"system","content":                                          "You are an AI assistant that categorizes questions and     notes submitted by patients to healthcare providers via an online portal     into Urgent and Non-Urgent messages. If the messages are medically urgent,     you should classify them as yes, otherwise no."},                              {"role":"user","content": "<MESSAGE_TEXT>"},                              {"role":"assistant","content": "<Yes|No>"}                              ]}


The prompt for Task B (Single vs. Multiple Category Detection) is presented in Listing 2.

**Listing 2.** Task B: Single vs. Multiple Categories prompt.
              {"messages": [                              {"role":"system","content":                                          "You are an AI assistant that categorizes questions and   notes submitted by patients to healthcare providers via an online portal   into whether the message involves Single or Multiple categories. If the   messages can be classified strictly into only one of the following   categories: Appointment, Referral Question, Request an Update to my Medical   Record, Visit Follow-Up Question, Question, Therapy Question, Refills,   Other, Test Results Question, and Prescription Question then it is a single   category message so your response should be as no, otherwise yes, if it is   a multiclass message."},                              {"role":"user","content": "<MESSAGE_TEXT>"},                              {"role":"assistant","content": "<Yes|No>"}                              ]}


The complete multi-label message classification prompt used for Task C is shown in Listing 3.

**Fine-Tuned LLMs**

For the fine-tuned condition, we adapted GPT-4o, BioBERT, and ClinicalBERT using a de-identified, clinician-annotated hospital dataset. Each training instance was serialized into a structured chat-style JSONL tuple [system, user, assistant], with assistant outputs constrained to the exact label set (e.g., Yes/No). Fine-tuning was performed in a HIPAA-compliant Azure environment, enabling the models to better capture institution-specific terminology, communication styles, and subtle contextual cues in patient–provider interactions.

**Summary**

Overall, the three approaches provided a spectrum of trade-offs. The TF-IDF + Logistic Regression baseline offered a transparent and computationally inexpensive benchmark. Non-fine-tuned prompting enabled rapid deployment and adaptability with minimal data requirements. Fine-tuned LLMs, by contrast, specialized to the local dataset and achieved the strongest performance, particularly in urgency detection and distinguishing single from multiple intents. Taken together, these strategies demonstrate how combining simple baselines with both general-purpose and domain-adapted LLMs can yield a comprehensive evaluation framework for patient message classification.

**Listing 3.** Task C: Full Message Classification prompt.
              {"messages": [                              {"role":"system","content":                                          "Your task is to classify the following patient question  or note into the following structure:                                        1. Urgency: Determine whether the message is Urgent or   Non-Urgent.                                        2. Category Type: Identify whether the message involves a   Single or Multiple category                                        3. Relevant Categories: Classify the message into one or   more of these predefined categories: Appointment, Referral Question,   Request an Update to my Medical Record, Visit Follow-Up Question, Question,   Therapy Question, Refills, Other, Test Results Question, and Prescription   Question.                                          Guidelines:                                          If the message is vague or unrelated, classify it under   Other. Prioritize clarity and intent to determine medical urgency.                                          Include all applicable categories if a message fits more   than one class.                                          Output Format: Urgency: [Urgent/ Non-Urgent], Category   Type: [Single/Multiple], Classes:  [List of Relevant Categories]"},                              {"role":"user","content": "<MESSAGE_TEXT>"},                              {"role":"assistant","content": "<row[’Combined_Output’]>"}                              ]}


### 3.4. Model Comparison

To evaluate the effectiveness of different LLMs in hospital message classification, we tested three key models, each with a distinct pretraining focus:

- **BioBERT** [11]—A biomedical domain adaptation of BERT, pre-trained on PubMed abstracts and PMC full-text articles. It has been widely used in medical NLP tasks such as named entity recognition and relation extraction.
- **ClinicalBERT** [12]—A model adapted from BERT, further pre-trained on MIMIC-III clinical notes, making it specialized for handling electronic health record (EHR) data.
- **GPT-4o** (OpenAI, 2024) [22]—The latest multimodal generative model capable of understanding and processing text with a broader, generalized training dataset, spanning medical, technical, and conversational domains.

**Figure 6:**
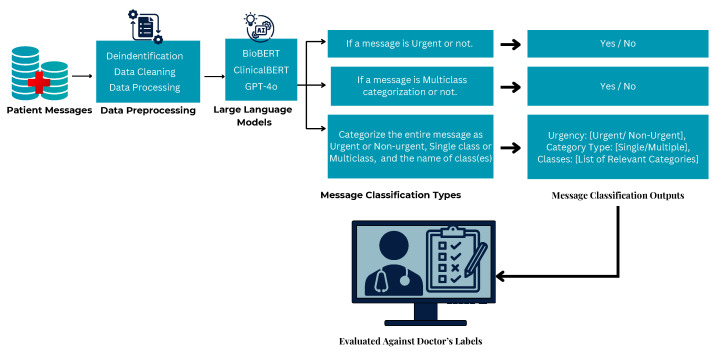
Baseline non-fine-tuned message classification by BioBERT, ClinicalBERT, and GPT-4o in a HIPAA-compliant environment.

**Figure 7:**
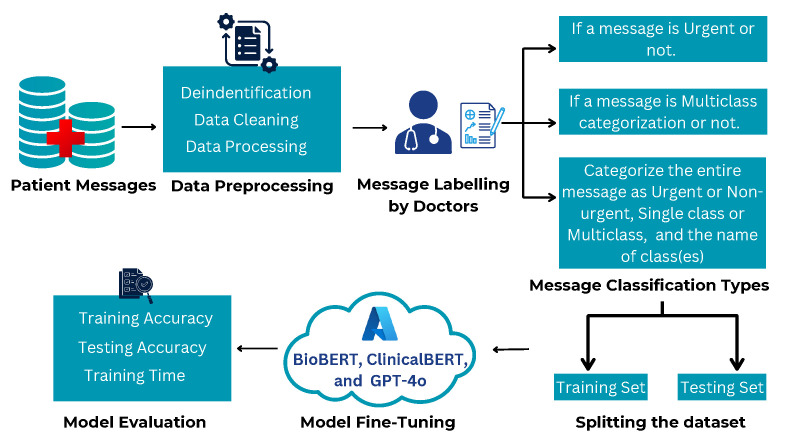
Fine-tuned message classification by BioBERT, ClinicalBERT, and GPT-4o in a HIPAA compliant environment.

Each model was tested with and without fine-tuning as illustrated in Figure 6 and Figure 7. This approach was chosen to assess their out-of-the-box capabilities in classifying real-world outpatient messages. BioBERT, ClinicalBERT and GPT-4o were fine-tuned within the hospital’s secure Azure OpenAI cloud as shown in Figure 7.

#### Factors Affecting Model Performance

Despite being trained on domain-specific data, BioBERT and ClinicalBERT struggled with multi-label classification. Several factors likely contributed to this outcome:

- **Pretraining Focus:** BioBERT was trained mainly for biomedical literature processing, making it effective for medical terminology but less adaptable to informal, multi-intent patient messages.
- **Data Domain Limitations:** ClinicalBERT was trained on structured EHR notes, which differ significantly from the short, often unstructured nature of patient communication in hospital portals.
- **Contextual Understanding:** Both models rely on BERT-style masked language modeling, which, while effective for extraction-based NLP tasks, may be less suited for complex, multi-class classification without additional fine-tuning.

In contrast, GPT-4o significantly outperformed both models in the few-shot setting, achieving urgency classification accuracy as high as 90% and full message classification accuracy around 24%. GPT-4o’s stronger performance can be attributed to its larger and more diverse training corpus, its multimodal capabilities, and its ability to generalize well across domains without requiring domain-specific pre-training.

### 3.5. Fine-Tuning and Non-Fine-Tuning Approaches

GPT-4o was tested with and without fine-tuning. Fine-tuned LLMs achieved over 90% accuracy across categories, outperforming non-fine-tuned models. Few-shot prompting improved contextual understanding and classification consistency. Fine-tuned GPT-4 demonstrated superior performance in multi-category classification tasks.

### 3.6. Fine-Tuning Setup

We fine-tuned GPT-4o, BioBERT, and ClinicalBERT with a learning rate of $2\times 10^{-5}$, batch size 5, and 3 epochs using AdamW. Input sequences were padded or truncated to a maximum of 256 tokens. All training and evaluation ran on Azure AI Foundry using serverless compute; the service abstracts the underlying hardware, so specific CPU/GPU details are not exposed to customers.

### 3.7. Evaluation Metrics and Validation Protocol

To improve statistical rigor and reduce sensitivity to a single split, we evaluate all models with **stratified *k*-fold cross-validation** (*k* = 10) on the labeled dataset. For each fold, the model is trained on k−1 folds and evaluated on the held-out fold; we report the mean test accuracy across fold.

## 4. Results and Analysis

### 4.1. Performance Metrics and Their Significances

In this section, we are going to discuss the used performance metrics in evaluating the comparative performance of the studied LLM models. A confusion matrix is a tool used to measure the performance of classification models by comparing actual vs. predicted values. Each entry in the matrix represents the count of predictions falling into four key categories:

- **True Positives (TP)**: Correctly predicted positive cases (e.g., urgent messages classified as urgent).
- **True Negatives (TN)**: Correctly predicted negative cases (e.g., non-urgent messages classified as non-urgent).
- **False Positives (FP)**: Incorrectly predicted positive cases (e.g., non-urgent messages classified as urgent), also known as type I error.
- **False Negatives (FN)**: Incorrectly predicted negative cases (e.g., urgent messages classified as non-urgent), also known as type II error.

**Figure 8:**
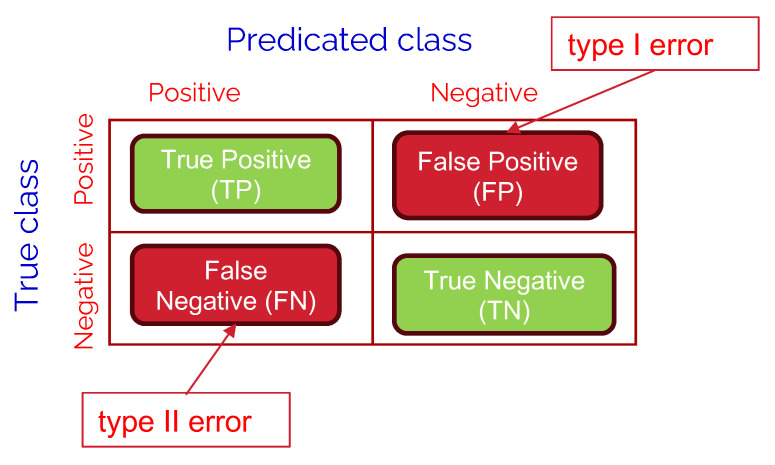
A typical structure of a binary classification confusion matrix.

Referring to the binary classification confusion matrix as shown in Figure 8, we define the following performance metrics:

- **Precision:**
  (1) $$\mathrm{Precision}=\frac{TP}{TP+FP}$$
  Precision represents the proportion of correctly predicted positive cases (e.g., urgent messages) out of all cases predicted as positive. A high precision value indicates that the model is good at avoiding false alarms (lower FP, namely, lower type I error), which is essential in scenarios where misclassification can result in unnecessary interventions or inefficiencies.
- **Recall:**
  (2) $$\mathrm{Recall}=\frac{TP}{TP+FN}$$
  Recall, also known as **sensitivity** or **true positive rate**, measures how well the model identifies actual positive cases. In the context of urgent messages, a higher recall means fewer critical cases are missed (lower FN, namely, lower type II error), ensuring that patients requiring immediate attention receive the necessary care.
- **F1-score:**
  (3) $$F1-\mathrm{Score}=2\times \frac{\mathrm{Precision}\times \mathrm{Recall}}{\mathrm{Precision}+\mathrm{Recall}}$$
  F1-score is the harmonic mean of precision and recall, providing a balanced evaluation of the model’s performance when both false positives and false negatives need to be minimized.
- **Accuracy:**
  (4) $$\mathrm{Accuracy}=\frac{TP+TN}{TP+TN+FP+FN}$$
  Accuracy gives an overall measure of correctness but can be misleading in imbalanced datasets where one class dominates.

### 4.2. Performance Evaluation

To evaluate the effectiveness of various LLMs in classifying outpatient messages, we first conducted experiments under a few-shot setting, followed by fine-tuning. As a benchmark, we implemented a TF-IDF + Logistic Regression model trained on the same dataset to provide a lightweight, interpretable baseline for comparison. Model performance was assessed across three dimensions—urgency classification, multi-class accuracy, and full message classification accuracy—the latter representing the combined ability to perform all classification tasks simultaneously.

Table 2 reports the performance of the baseline model. While Logistic Regression provided a reasonable starting point for urgency classification, its performance on single/multiple category prediction was notably lower, underscoring the added value of LLMs for more nuanced classification tasks. This comparison helps justify the need for large-scale language models and fine-tuning for robust message understanding in clinical workflows.

Compared to this baseline, fine-tuned GPT-4o achieved gains of over **21% in urgency classification** and nearly **28% in single/multiple classification accuracy**, highlighting the significant improvement provided by modern LLMs over traditional feature-based models.

**Figure 9:**
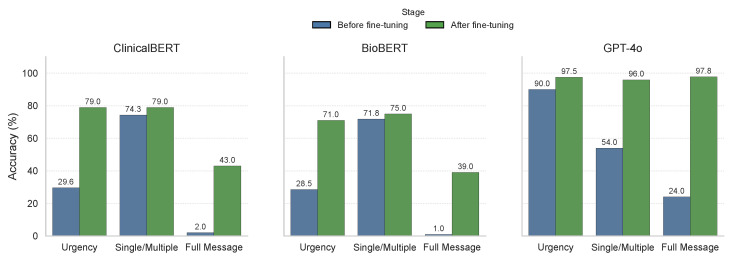
Comparison of different LLMs with and without fine-tuning. GPT-4o significantly outperformed BioBERT and ClinicalBERT across all classification tasks, highlighting its superior generalization abilities.

Figure 9 presents a comparative analysis of BioBERT, ClinicalBERT, and GPT-4o in a few-shot non-finetuned classification setting. While BioBERT and ClinicalBERT were designed for domain-specific biomedical and clinical tasks, their performance was lower in urgency and full message categorisation accuracy due to the informal nature of patient messages. In contrast, as shown in Table 3, GPT-4o demonstrated higher accuracy across urgency and category classification.

Table 3 quantifies the performance gaps observed in Figure 9. BioBERT and ClinicalBERT performed relatively well in category type classification i.e., single or multiple, but struggled with urgency categorization and full message classification. These findings suggest that while domain-specific models are effective for structured medical data, they require additional fine-tuning to adapt to informal and patient-centered communication.

### 4.3. K-Fold Cross-Validated Results

Table 3 summarizes few-shot (no fine-tuning) accuracy, and Table 4 reports 10-fold cross-validated accuracy after fine-tuning. These results show substantial gains from fine-tuning for all models, with the largest improvements for GPT-4o across all tasks.

**Analysis:** Fine-tuning consistently improves performance for all architectures. GPT–4o attains the highest cross-validated accuracy on *Urgency* (97.50%), *Single/Multiple* (95.95%), and *Full Message* (97.79%), indicating strong generalization across folds. ClinicalBERT and BioBERT benefit from fine-tuning but remain lower on full message classification, suggesting difficulty handling informal, multi-intent phrasing compared with GPT-4o.

### 4.4. Impact of Fine-Tuning

All three models, GPT-4o, BioBERT, and ClinicalBERT were fine-tuned on the outpatient messaging dataset within a secure HIPAA-compliant Azure OpenAI environment. Fine-tuning consistently improved classification performance across urgency, single/multiple categorization, and full message classification for every model. The most pronounced gains were observed for GPT-4o, which improved from 90% to 97.50% in urgency classification and from 54% to 95.95% in single/multiple classification, a substantial gain of over 41%.

When compared to the TF-IDF + Logistic Regression baseline, the fine-tuned models delivered exceptional performance improvements—**+21% in urgency classification** and **+28% in single/multiple classification accuracy** for GPT-4o—with BioBERT and ClinicalBERT also achieving double-digit improvements over baseline. GPT-4o further outperformed BioBERT and ClinicalBERT by over **+18% in urgency accuracy** and more than **+50% in full message classification**, underscoring its superior ability to generalize across diverse and informal patient messages.

Table 4 summarizes the fine-tuned results across all three tasks, clearly demonstrating that while BioBERT and ClinicalBERT benefited from fine-tuning, GPT-4o consistently achieved the highest performance across all evaluation metrics. Across ten-fold cross-validation, the standard deviation of model accuracy remained low: within ±1.5% for GPT-4o and within ±2.1% for BioBERT and ClinicalBERT. These values indicate high stability and reproducibility of the results.

### 4.5. Implications for Outpatient Message Classification

The results of this study hold several practical implications for real-world outpatient message management. From a clinical operations perspective, maintaining a high recall rate for urgent messages is paramount, as missing even a small number of critical cases could compromise patient safety. Conversely, achieving high precision in non-urgent message detection is equally valuable because it reduces unnecessary escalations and minimizes the cognitive and administrative workload on providers. Striking the right balance between recall and precision is therefore central to achieving an effective triage system. In multi-label classification settings, where a single message may belong to more than one category, the F1-score serves as a robust indicator of model reliability because it jointly captures the trade-off between false positives and false negatives. Overall, these findings underscore the importance of model interpretability and reliability when integrating automated systems into patient–provider communication workflows.

### 4.6. Key Observations and Insights

The study provides several insights into the design and behavior of LLMs for healthcare message classification. Although the three subtasks—urgency detection, intent identification, and category classification—can theoretically be implemented as sequential stages, we treated them as independent experiments to mirror the modular structure of real-world triage systems. Conducting full classification as a single step allowed the models to capture interdependencies between urgency and message content, reducing potential error propagation between subtasks. This design choice aligns closely with clinical workflows, where messages are assessed holistically rather than through a rigid, stepwise process.

Among the evaluated models, BioBERT and ClinicalBERT demonstrated strong performance in urgency classification but comparatively lower accuracy in multi-label categorization, reflecting their specialization in structured biomedical narratives rather than informal, conversational patient communications. GPT-4o, by contrast, exhibited superior few-shot and fine-tuned performance, highlighting its versatility and ability to generalize across diverse linguistic contexts. Fine-tuning all three models enabled a fair comparative assessment, revealing GPT-4o’s distinct advantage in capturing multi-intent and contextually nuanced message types. Importantly, all experiments were conducted within a HIPAA-compliant Azure OpenAI environment, ensuring privacy-preserving fine-tuning without compromising data security. Together, these findings emphasize both the promise and practical challenges of integrating LLMs into clinical communication pipelines.

## 5. Discussion

Large Language Models (LLMs) demonstrated high accuracy and adaptability for outpatient message classification, thereby reducing manual effort and improving workflow efficiency. Challenges remain in computational costs, bias mitigation, and domain adaptation. Ethical considerations such as privacy, fairness, and transparency are essential for deployment. Our findings align with recent studies such as MedAlpaca [13], GatorTron [14], and BioGPT [23], which also demonstrated strong generalization capacity of large transformer models for clinical NLP. Compared with these, our study uniquely focuses on outpatient message triage and directly integrates fine-tuning under HIPAA-compliant infrastructure, bridging theoretical advances with deployable clinical applications.

### 5.1. Strengths

The proposed framework shows several notable strengths that reinforce its applicability to real-world outpatient message management. First, the fine-tuned GPT-4o model achieved consistently high accuracy across all classification subtasks, including urgency detection, multi-class labeling, and multi-label categorization. This performance translated into a meaningful reduction in manual triage workload for clinical staff, as the model could reliably differentiate between routine and urgent cases. In addition, the system proved capable of handling varied message styles and multi-intent content, reflecting the diverse linguistic patterns common in patient–provider communication. Together, these results highlight the potential of large language models to improve workflow efficiency while preserving clinical reliability and interpretability.

### 5.2. Limitations

Several limitations should be acknowledged. The dataset was relatively small and derived from a single institution, which may constrain linguistic diversity and generalizability. Because each message was annotated by physicians, annotation throughput was limited; however, this ensured high labeling reliability. Computational resources also restricted the exploration of large-scale hyperparameter tuning. To mitigate potential overfitting and sample sensitivity, ten-fold cross-validation was applied to average results across multiple splits. Furthermore, due to institutional data restrictions, it was not possible to conduct formal statistical hypothesis testing between models. Instead, model stability was assessed through fold-to-fold variance, which remained within ±1.5% for GPT-4o, supporting the robustness of the observed performance. Future work will expand the sample size, include additional hospital systems, and incorporate comprehensive statistical validation to confirm the generalizability of these findings.

### 5.3. Ethical Considerations

All patient messages used in this study were fully de-identified in accordance with HIPAA and institutional privacy policies. Personally identifiable information and protected health information were removed to ensure confidentiality. Although this approach preserved privacy, it also limited the ability to analyze potential performance variations across demographic or linguistic subgroups. We therefore identify this as an important area for future research. Addressing bias and fairness will involve strategies such as bias-aware data augmentation, synthetic message generation, to represent under-represented communication styles, and external validation using multi-institutional datasets. These measures will support the equitable and responsible deployment of language models in clinical environments.

### 5.4. Practical Applications

The findings of this study have several practical implications for healthcare operations. Integrating LLM-based triage systems into hospital communication platforms can substantially improve message-handling efficiency and reduce administrative burden on providers. Automated flagging of urgent clinical messages can facilitate rapid intervention, while structured categorization of routine queries supports faster routing and response generation. Beyond operational benefits, such systems can also enhance patient experience by ensuring timely acknowledgment of messages and providing automated responses for common non-urgent requests. Ultimately, these applications demonstrate the feasibility of safely incorporating LLM-driven automation into real-world healthcare workflows under appropriate oversight and privacy safeguards. As a practical example, the fine-tuned GPT-4o model could be integrated within the hospital’s electronic message triage system. Incoming outpatient messages would be automatically classified by urgency and intent, allowing critical cases (e.g., acute symptom reports) to be flagged for immediate review, while administrative requests (e.g., appointment scheduling) are routed to support staff. This workflow reduces response times and clinician workload while maintaining HIPAA compliance and operational efficiency.

## 6. Conclusions and Future Work

This study evaluated GPT-4o, BioBERT, and ClinicalBERT for outpatient message classification across urgency, multi-class, and multi-label tasks, using a TF-IDF + Logistic Regression (LR) model as a baseline. While LR provided a reasonable starting point for urgency classification, its performance on single/multiple categorization was notably lower, underscoring the need for more sophisticated approaches. All three LLMs demonstrated clear improvements over the LR baseline, with GPT-4o achieving the highest overall performance, particularly in multi-intent categorization. BioBERT and ClinicalBERT also performed competitively in urgency detection when fine-tuned, highlighting that domain-specific models still offer value for structured tasks.

Fine-tuning was conducted with **10-fold cross-validation** to improve generalizability and reduce overfitting risks. This process consistently improved results for all three models, with GPT-4o showing the largest absolute gains, confirming the value of adapting LLMs to domain-specific data. The results highlight that:

- LLMs substantially outperform traditional baselines. Fine-tuned GPT-4o improved urgency classification by over 21% and single/multiple categorization by nearly 28% relative to the LR model.
- GPT-4o’s broad pretraining provides a distinct advantage in processing informal, multi-intent patient messages, outperforming domain-specific BioBERT and ClinicalBERT on full message classification.
- Fine-tuning with 10-fold cross-validation enabled a robust, fair comparison, ensuring results did not depend on a single train-test split and clarifying the trade-offs between general-purpose and domain-specific LLMs for healthcare applications.

Future work will include:

- Real-time deployment and monitoring of LLM-powered triage systems in clinical and administrative workflows.
- Multi-modal integration of structured EHR data with unstructured messages for richer context-aware classification.
- Scaling datasets across institutions and patient populations to improve generalization, fairness, and robustness.
- Exploring cost–benefit trade-offs between LLM fine-tuning and lighter-weight baselines such as LR for resource-constrained deployments, where simpler models may still offer acceptable performance.

In conclusion, our research contributes theoretically, methodologically, and practically. Theoretically, it demonstrates that general-purpose LLMs can equal or surpass domain-specific models in clinical text classification. Methodologically, it presents a reproducible framework integrating fine-tuning, few-shot prompting, and cross-validation within a secure healthcare environment. Practically, it delivers a deployable, privacy-compliant message triage system aligned with actual hospital workflows. Future work will expand validation across multiple institutions, incorporate multimodal EHR data, and analyze deployment efficiency and cost trade-offs against more future emerging LLMs model.

## Figures and Tables

**Table 1:** Message category example.

Message	Urgency	Multiple Category	Category
I’m feeling chest tightness and need to know if I should go to the ER or wait.	Urgent	Single	Question
Please refill my blood pressure meds and let me know if Dr. Smith reviewed my blood test.	Non-Urgent	Multiple	Refills; Test Results
Can I schedule my next follow-up visit for a diabetes check-up?	Non-Urgent	Single	Appointment

**Table 2:** TF-IDF + Logistic Regression Baseline Accuracy (%).

Task	Accuracy
Urgency (Binary)	76.19
Single/Multiple	67.86

**Table 3:** Without fine-tuning accuracy (%).

Model	Urgency	Single/Multiple	Full Message
ClinicalBERT	29.64	74.29	2.00
BioBERT	28.50	71.79	1.00
GPT–4o	90.00	54.00	24.00

**Table 4:** After fine-tuning: 10-fold cross-validated accuracy (%). Values are fold means.

Model	Urgency	Single/Multiple	Full Message
ClinicalBERT	79.00	79.00	43.00
BioBERT	71.00	75.00	39.00
GPT–4o	97.50	95.95	97.79

## Data Availability

The data presented in this study are unavailable on request from the corresponding author. The data are not publicly available due to hospital confidentiality policies.

## Abbreviations

The following abbreviations are used in this manuscript:

HIPAA	The Health Insurance Portability and Accountability Act of 1996 in the Unites States.
EHR	Electronic Health Record
LLM	Large Language Model
ChatGPT	ChatGPT is a one kind of LLM [22]
BioBERT	BioBERT is a one of LLM [11]
ClinicalBERT	ClinicalBERT is a one kind of LLM [12]

## References

[1] Consulting L. (2025). Tapping into New Potential: Realising the Value of Data in the Healthcare Sector. https://www.lek.com/insights/hea/eu/ei/tapping-new-potential-realising-value-data-healthcare-sector.

[2] Jensen P.B., Jensen L.J., Brunak S. (2012). Mining Electronic Health Records: Towards Better Research Applications and Clinical Care. Nat. Rev. Genet..

[3] Shah A., Chen B. (2024). Optimizing Healthcare Delivery: Investigating Key Areas for AI Integration and Impact in Clinical Settings.

[4] Hond A.A.H.d., Leeuwenberg A.M., Hooft L., Kant I.M.J., Nijman S.W.J., van Os H.J.A., Aardoom J.J., Debray T.P.A., Schuit E., van Smeden M. (2022). Guidelines and quality criteria for artificial intelligence-based prediction models in healthcare: A scoping review. NPJ Digit. Med..

[5] Paucar E., Paucar H., Paucar D., Paucar G., Sotelo C. (2024). Artificial intelligence as an innovation tool in hospital management: A study based on the sdgs. J. Lifestyle SDGs Rev..

[6] Lorencin I., Tanković N., Etinger D. (2025). Optimizing healthcare efficiency with local large language models. Intelligent Human Systems Integration (IHSI 2025): Integrating People and Intelligent Systems.

[7] Nashwan A., Abujaber A. (2023). Harnessing the power of large language models (llms) for electronic health records (ehrs) optimization. Cureus.

[8] Smith J., Doe J. (2019). Automated Classification of Clinical Text using Machine Learning. J. Med. Inform..

[9] Jones R., White S. (2020). Medical Text Classification Using Deep Learning Techniques. Artif. Intell. Med..

[10] Brown E., Taylor M. (2021). Deep Learning Models for Electronic Health Record Classification. IEEE Trans. Biomed. Eng..

[11] Lee J., Yoon W., Kim S., Kim D., So J., Kang H. (2020). BioBERT: A Pre-trained Biomedical Language Representation Model for Biomedical Text Mining. Bioinformatics.

[12] Alsentzer E., Murphy J.R., Boag W., Weng W.H., Jin H., Naumann T., McDermott M.B. (2019). Publicly Available Clinical BERT Embeddings. arXiv.

[13] Han T., Adams L.C., Papaioannou J.M., Grundmann P., Oberhauser T., Löser A., Truhn D., Bressem K.K. (2023). MedAlpaca: An Open-Source Collection of Medical Conversational AI Models and Training Data. arXiv.

[14] Yang X., Chen A., PourNejatian N., Shin H.C., Smith K.E., Parisien C., Compas C., Martin C., Flores M.G., Zhang Y. (2022). GatorTron: A Large Clinical Language Model to Unlock Patient Information from Unstructured Electronic Health Records. arXiv.

[15] Achiam J., Adler S., Agarwal S., Ahmad L., Akkaya I., Aleman F.L., Almeida D., Altenschmidt J., Altman S., OpenAI (2024). GPT-4 Technical Report. arXiv.

[16] Wu P., Kumar A., Smith L. (2023). Comparing Domain-Specific and Generalist Large Language Models for Medical Text Classification. J. Artif. Intell. Healthc..

[17] OpenAI (2024). GPT-4o: OpenAI’s Latest Multimodal Model. https://platform.openai.com/docs/models/gpt-4o.

[18] Qiu J., Jiang M., Zhang T. (2023). Large Language Models for Medical Applications: Challenges and Future Directions. IEEE Trans. Neural Netw. Learn. Syst..

[19] Zhang H., Liu X., Peng J. (2023). Generative AI for Clinical Report Generation: A Systematic Review. J. Med. Inform..

[20] Dai X., Qian Y., Liu F. (2023). Automating Healthcare Administrative Workflows with Large Language Models. Artif. Intell. Med..

[21] HIPAA for Professionals. https://www.hhs.gov/hipaa/for-professionals/index.html.

[22] GPT-4o. https://openai.com/index/hello-gpt-4o/.

[23] Luo R., Sun L., Xia Y., Qin T., Zhang S., Liu T.Y. (2022). BioGPT: Generative pre-trained transformer for biomedical text generation and mining. Briefings Bioinform..

